# Wound healing: insights into autoimmunity, ageing, and cancer ecosystems through inflammation and IL-6 modulation

**DOI:** 10.3389/fimmu.2024.1403570

**Published:** 2024-11-29

**Authors:** Lukáš Lacina, Michal Kolář, Lucie Pfeiferová, Peter Gál, Karel Smetana

**Affiliations:** ^1^ Institute of Anatomy, First Faculty of Medicine, Charles, University, Prague, Czechia; ^2^ BIOCEV, First Faculty of Medicine, Charles University, Vestec, Czechia; ^3^ Department Dermatovenereology, First Faculty of Medicine, Charles University and General University Hospital, Prague, Czechia; ^4^ Laboratory of Genomics and Bioinformatics, Institute of Molecular Genetics of the Czech Academy of Sciences, Prague, Czechia; ^5^ Department of Pharmacology, Faculty of Medicine, Pavol Jozef Šafárik University in Košice, Košice, Slovakia; ^6^ Department of Biomedical Research, East-Slovak Institute of Cardiovascular Diseases Inc., Košice, Slovakia; ^7^ Prague Burn Centre, Third Faculty of Medicine, Charles University and University Hospital Královské Vinohrady, Prague, Czechia; ^8^ Department of Pharmacognosy and Botany, Faculty of Pharmacy, Comenius University, Bratislava, Slovakia

**Keywords:** wound healing, granulation tissue, myofibroblasts, cancer-associated fibroblasts, IL-6

## Abstract

Wound healing represents a complex and evolutionarily conserved process across vertebrates, encompassing a series of life-rescuing events. The healing process runs in three main phases: inflammation, proliferation, and maturation/remodelling. While acute inflammation is indispensable for cleansing the wound, removing infection, and eliminating dead tissue characterised by the prevalence of neutrophils, the proliferation phase is characterised by transition into the inflammatory cell profile, shifting towards the prevalence of macrophages. The proliferation phase involves development of granulation tissue, comprising fibroblasts, activated myofibroblasts, and inflammatory and endothelial cells. Communication among these cellular components occurs through intercellular contacts, extracellular matrix secretion, as well as paracrine production of bioactive factors and proteolytic enzymes. The proliferation phase of healing is intricately regulated by inflammation, particularly interleukin-6. Prolonged inflammation results in dysregulations during the granulation tissue formation and may lead to the development of chronic wounds or hypertrophic/keloid scars. Notably, pathological processes such as autoimmune chronic inflammation, organ fibrosis, the tumour microenvironment, and impaired repair following viral infections notably share morphological and functional similarities with granulation tissue. Consequently, wound healing emerges as a prototype for understanding these diverse pathological processes. The prospect of gaining a comprehensive understanding of wound healing holds the potential to furnish fundamental insights into modulation of the intricate dialogue between cancer cells and non-cancer cells within the cancer ecosystem. This knowledge may pave the way for innovative approaches to cancer diagnostics, disease monitoring, and anticancer therapy.

## Introduction

Globally, the elderly population is experiencing significant growth, attributed to enhanced management of chronic diseases (1). In 2004, there were 461 million individuals aged 65 and older, and projections suggest this number will increase to 2 billion by 2050, posing unprecedented challenges for healthcare planning and delivery (2). Recognising ageing as a key factor in chronic conditions (3), the growing elderly population’s socio-economic impact has forced research to focus on the aspects of extending human health span (4).

Wound healing is a fundamental biological response, an intricate orchestration of events, offering a unique lens through which we can unravel the complexities of autoimmune disorders, ageing-related conditions, and the intricate landscape of cancer. The present review aims to underscore the important role of wound healing as a model for understanding these overarching biological processes. Autoimmunity, ageing, and cancer, seemingly different biological events, reveal interconnected threads that converge in the epicentre of wound healing. Central to this convergence is the orchestrating influence of inflammation, with interleukin-6 (IL-6) emerging as a key regulatory player. The structural parallels between tumours and wound healing have been recognised for decades, notably illustrated in the seminal work by Dvorak in 1986 (5). Recent insights emphasise the striking resemblance between granulation tissue, pannus-like tissue from chronic inflammation (such as progressive arthritis), and tissues attacked by viral infections, with the solid cancer stroma (6). These pathologies result in fibrotic tissue, also observed in conditions such as tissue fibrosis in the lungs of COVID-19 patients or the stroma of pancreatic ductal adenocarcinoma (PDAC) (6, 7).

We will delve into the stages of wound healing, exploring the dynamic interplay of cellular and molecular components. By unravelling the parallels between wound healing and autoimmune responses, investigating the impact of ageing on tissue regeneration, and scrutinising the intricate relationship between wound healing and cancer ecosystems, we aspire to elucidate the profound implications that a comprehensive understanding of wound healing holds for these complex biological phenomena. The spotlight on inflammation, especially IL-6, serves as the central key point, guiding our exploration where wound healing serves not only as a metaphor but also as a model for broader biological processes.

## Wound healing

**Figure 1:**
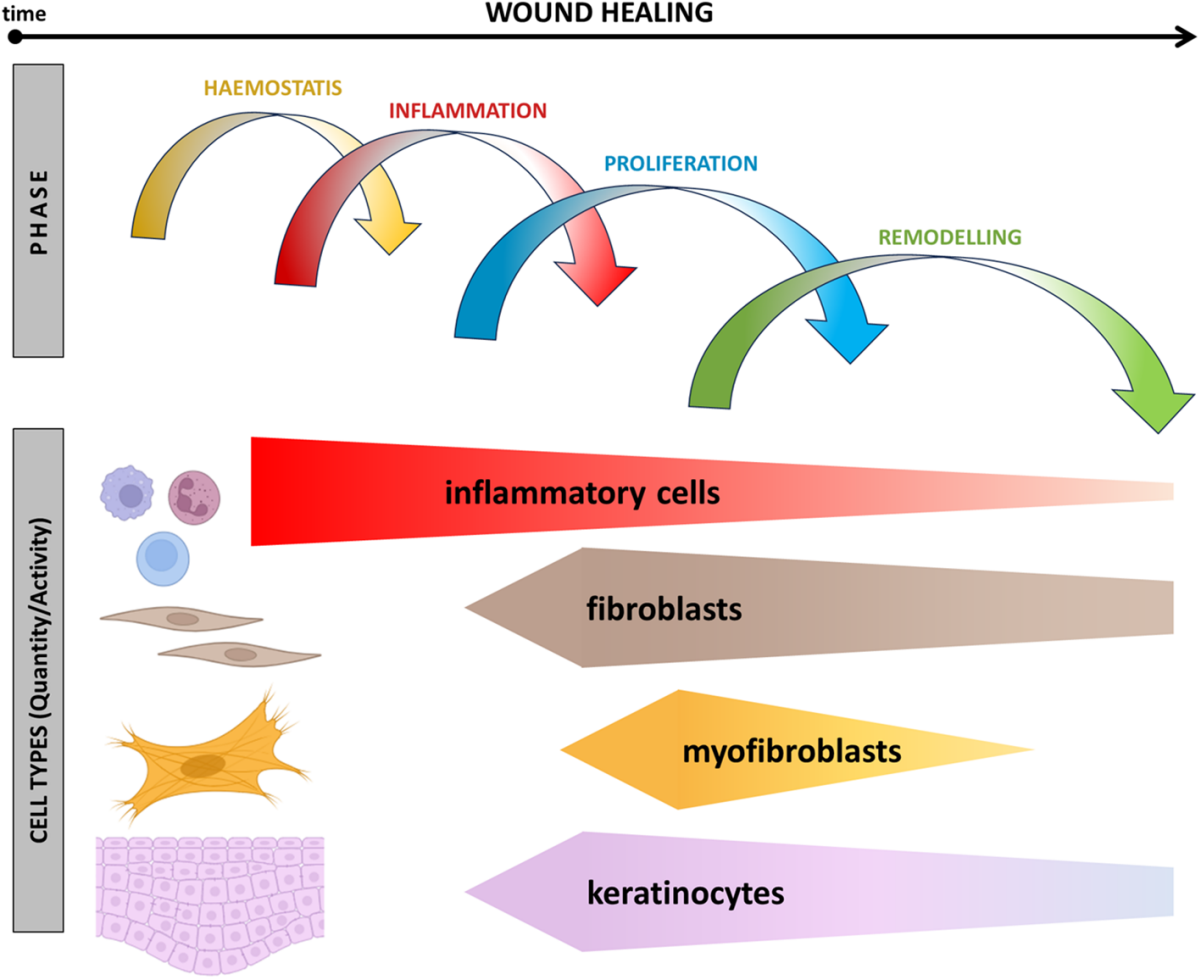
Schematic presentation of events associated with wound healing over time. The relationship between the presence of inflammatory cells, fibroblasts, myofibroblasts, and reepithelisation across different phases of wound healing is shown.

Wound healing is a dynamic process influenced by various factors, including the size and extent of the wound, microbial contamination, and the overall health and age of the individual. Failure to navigate this intricate process can result in chronic wounds or formation of hypertrophic/keloid scars (8–10). An intriguing aspect lies in the regenerative capacity of the interfollicular epidermis in humans, capable of full regeneration. In contrast, the repair of the dermal architecture in humans is a more intricate process leading to scar formation. This dermal repair unfolds through three/four distinct phases: blood clotting/haemostasis (potentially part of the inflammatory response), inflammation, proliferation, and maturation/remodelling. Epidermal regeneration occurs concurrently with dermal healing, and the restoration of the barrier integrity is completed during the process of re-epithelisation, while dermal remodelling continues for several weeks or months after (11). Despite the complexity and precision inherent in wound healing, this orchestrated process is not immune to interruptions and failures. Such disruptions can result in the development of non-healing/chronic wounds or pathological scars, underscoring the delicate balance required for successful tissue repair and regeneration (8). The vulnerability of the wound healing process to deviations emphasises the need for a nuanced understanding of its intricacies to develop effective interventions and therapies. The key events in the wound healing sequence have been described in detail previously (8, 12) and are summarised in **Table 1** and **Figure 1**.

**Table 1:** Wound healing (humans).

**Phase 1** Inflammation	After haemostasis, the wound bed is infiltrated by immune cells from both innate and adaptive immunity, including neutrophils, macrophages, and lymphocytes. This infiltration aims to prevent wound contamination by pathogens and facilitate phagocytosis of the tissue debris. Additionally, these immune cells produce a range of inflammation-supporting growth factors, cytokines, and chemokines.
**Phase 2** Proliferation	Fibroblasts and other precursors are recruited to the wound, where they proliferate and generate an extracellular matrix, along with various active factors (including inflammation-supporting factors as observed in Phase 1). This phase is characterised by maximal development of granulation tissue, accompanied by continuation of the inflammation. It stimulates angiogenesis of the wound bed and the process of re-epithelisation, involving epithelial-mesenchymal transition, migration of epithelial cells, and their proliferation. Additionally, many fibroblasts transform into myofibroblasts, also contributing to the wound contraction.
**Phase 3** Remodelling	Fibroblasts produce both extracellular matrix and proteolytic enzymes responsible for the degradation of extracellular matrix, leading to reduced scar (fibrosis) formation.

### Inflammatory phase

Traumatic tissue injury often initiates with haemorrhage, leading to the formation of a stable haemostatic clot composed of polymerised fibrin. This essential process, known as haemostasis, is crucial in preventing major blood loss. The resulting coagulum, which temporarily fills the wound defect and forms a protective crust, serves as a provisional barrier, safeguarding deeper tissues. Both platelets and clotting factors participate in haemostasis, with the intrinsic and extrinsic pathways distinguished by the upstream factors that activate the coagulation cascade, ensuring swift establishment of a temporary protective barrier (12). Subsequent to haemostasis, vessel dilatation and permeabilisation facilitate the migration of leucocytes to the injury site, marking the onset of the inflammatory phase. The primary goal of infiltrating leucocytes is to eliminate pathogens, resolve inflammation, and clear necrotic cells/tissue (13). Key immune cell players include granulocytes (neutrophils and eosinophils), macrophages, mast cells, natural killer (NK) cells, B lymphocytes, and various T lymphocyte subsets. Neutrophils, the initial and predominant immune cells at the wound site, play a pivotal role in the removal of debris and pathogens (14). Macrophages play a central role throughout all phases of wound healing. M1-activated macrophages are crucial for orchestrating the onset of the inflammatory phase, while their reprogramming into M2-polarised macrophages is essential for resolving inflammation and supporting the subsequent phases of proliferation and remodelling. The ultimate functional and morphologic outcome of wound healing relies heavily on the successful resolution of inflammation (15). Persistent inflammation may disrupt keratinocyte and fibroblast differentiation, potentially leading to excessive scarring and, in severe cases, formation of hypertrophic/keloid scars (16).

To orchestrate the inflammatory activity across the wound site, immune cells release a diverse array of bioactive factors. These encompass transforming growth factor (TGF)-β, tumour necrosis factor (TNF)-α, interferon (IFN)-γ, vascular endothelial growth factor A (VEGFA), basic fibroblast growth factor (bFGF), interleukins (IL-4, -6, -9 -13, -17, -23), chemokines (CXCL-8, CXCL-12), and numerous proteases (8). Notably, certain factors exhibit pleiotropy, being produced by multiple immune cell types. For instance, IL-6 is synthesised by neutrophils, macrophages, mast cells, and T lymphocytes. Similarly, members of the TGF-β family are widely produced by immune cells, influencing various cells in the wound microenvironment (WME) simultaneously. Moreover, these cytokines can influence local fibroblasts, keratinocytes, and endothelial cells. These cytokines can also enter systemic circulation, making them measurable indicators for monitoring large wound healing (17). Hyperactivation of the immune system, although rare, has been observed in large, infected wounds or extensive burn injuries, leading anecdotally to the so-called cytokine storm syndrome (18).

### Proliferation phase

Successful resolution of inflammation marks the transition to the proliferation phase, typically observed 2–4 days post-injury (19). Central to this phase is formation of granulation tissue, a provisional structure comprising persisting recruited inflammatory/immune cells, sprouting capillary endothelium, and fibroblasts actively engaged in extracellular matrix (ECM) production. Initially, fibroblasts generate a loose ECM, forming a temporary scaffold vital for the migration and proliferation of other cells involved in the healing process (20). Remarkably, dermal fibroblasts constitute a remarkably heterogeneous population of mesenchymal cells, with two principal subgroups - papillary and reticular - identified histologically. These subsets differ in proliferative capacity, wound contraction ability, and expression profiles. Recent single-cell sequencing has unveiled up to six functionally distinct subsets of dermal fibroblasts, among which the CD26-expressing population stands out as a key contributor to ECM production and, consequently, new tissue formation during the wound healing (21). The resulting scar’s characteristics are intricately linked to the fibroblast subset predominant at the wound site (22).

[note: Origin of fibroblasts - where do they come from? The intriguing diversity among fibroblasts may also stem from their developmental origin. In the craniofacial region, dermal fibroblasts may have a dual embryonic origin, originating from both cranial neural crest (facial fibroblasts) and cephalic mesoderm. Dorsal skin fibroblasts trace back to the somites, while those in the ventral flank and limb dermis arise from the lateral plate mesoderm (23). Notably, fibroblasts or fibroblast-like cells can emerge from various cell populations, including epithelial cells, through epithelial-to-mesenchymal transition (EMT) (24), epidermal stem cells (25), bone marrow-derived mesenchymal stem cells (26), and circulating fibrocytes (27). Additionally, endothelial-to-mesenchymal transition (EndMT) offers another pathway for the generation of fibroblast-like cells (28).]

Within the intricate landscape of fibroblasts, a distinctive subpopulation emerges, characterised by the active *ACTA2* gene, encoding α-smooth muscle actin. This distinctive fibroblast subpopulation plays a significant role in wound contraction, also facilitating the subsequent process of re-epithelisation (29, 30). While playing a central role in the wound contraction, myofibroblasts demonstrate a dual nature. In addition, these cells are potent producers of a diverse array of bioactive factors. Notably, among these factors are those with inflammation-supporting activity, such as IL-6 and IL-8, not only contributing to inflammation but also exhibiting pro-fibrotic effects. Multiple stimulators, including cytokines (TGF-β1), growth factors (CTGF, FGF, PDGF, IGF), and galectins (galectin-1), have been identified to regulate fibroblast and myofibroblast activities (31–35). TGF-β1, in particular, stands out as a recurrently reported critical factor driving fibroblast differentiation into myofibroblasts (36) (**Figure 1**). This intricate interplay between diverse fibroblast subsets and their regulatory factors forms the foundation for understanding the complexities of wound healing and scar formation.

Simultaneously with the proliferation phase, the critical process of angiogenesis unfolds, fostering the formation of new blood vessels to meet the escalating demands of rapidly proliferating cells (37). The vascular endothelial growth factor (VEGF) stands at the forefront of pro-angiogenic molecules during wound healing. Recognised as a vascular endothelial cell mitogen and a regulator of endothelial integrin expression during vessel sprouting, VEGF extends its influence as a chemokine for macrophages, activating them directly via the VEGF receptor (VEGFR) (38). Consequently, VEGF assumes the role of an indirect pro-inflammatory cytokine, potentially contributing to excessive scar formation.

Re-epithelisation stands as the ultimate process in the establishment of a protective barrier, shielding the body’s interior from potential microbial threats and minimising the loss of essential fluids and crystalloids (8). Keratinocytes, particularly those residing in the wound margins and hair follicles, emerge as a cellular base for re-epithelisation. This intricate process involves a harmonious interplay of biological events encompassing cell proliferation, epithelial-mesenchymal transition, and migration (39, 40). Underpinning re-epithelisation is the intrinsic genetic programme of epithelial cells, orchestrated in conjunction with the active involvement of granulation tissue cells and their secretory products that influence the epithelium (41). The interaction between the epithelium and granulation tissue cells is bidirectional, as the completion of wound closure marks the cessation of ECM, growth factor, cytokine, and chemokine production by granulation tissue cells, initiating the subsequent phase of scar remodelling (41, 6). This dynamic interplay ensures the seamless progression from wound closure to scar maturation, contributing to the overall integrity and functionality of the healed tissue.

### Maturation/Remodelling phase

Regeneration in postnatal mammals is a rare phenomenon, with the human liver being a notable exception. In the majority of tissues, wound healing culminates in scar formation. Following wound closure, a critical phase of remodelling ensues, predominantly impacting the granulation tissue. This intricate process necessitates the degradation of the ECM and its subsequent reconstruction in the scar. Proteolytic enzymes play a remarkable role in wound healing, specifically in the context of tissue remodelling (42, 43). The degradation of the non-matured ECM is instrumental in facilitating the establishment of the matured matrix, important for achieving the correct size of the scar while ensuring sufficient strength to prevent secondary wounding due to mechanical stress (8, 44). Any misstep in these interactions can result in suboptimal healing outcomes, with scars that may be inconspicuous in neonates but more prominent in adults (45). It has been suggested that neonatal fibroblasts preserve distinct properties of mesenchymal stem cells (46), accompanied by the deregulation of TGF-β signalling and low expression of the TGF-β II receptor (47). Despite being a well-documented clinical phenomenon, the complex mechanisms underlying the age-dependent variation in healing capacity remain a subject of ongoing exploration. As the granulation tissue formation gradually halts through cell apoptosis, the wound transforms into an almost avascular and acellular scar (8).

In cases of complicated wounds with prolonged inflammatory response, for example, due to contamination by microorganisms or debris, scarring can become intricate, potentially leading to hypertrophic or keloid scars (48, 49). There is evidence suggesting a genetic predisposition to keloid formation, with ethnicity also playing a role. The highest incidence of keloids is reported in Africa (5-10%), with Asia following closely (up to 1%) (50). However, our understanding of the genetics underlying keloid formation is still evolving, and further research is needed in this area (51). Keloid formation is a complex process, sharing similarities with tumour development, particularly in the context of stromal characteristics (52). Inflammation is a key player in the aetiopathogenesis of keloids and hypertrophic scars, evident in the infiltration of leucocytes into the scar tissue (8, 53, 54). This pro-inflammatory microenvironment supports the presence of myofibroblasts, aligning with their frequent occurrence. In contrast to tumour stroma, keloid scars lack elastin and hyaluronic acid (55), further distinguishing their unique composition. Unravelling the intricate mechanisms underlying keloid formation holds promise for developing targeted interventions to modulate scar outcomes and improve patient outcomes.

## Organ fibrosis

Connective tissue, containing the intricate molecules of the ECM, is a universal component found in all organs. The deposition of ECM plays an irreplaceable role in the healing process. However, an imbalance between deposition and degradation can lead to organ fibrosis, resulting in scar formation, and significantly affecting the organ function (56). Inflammation further contributes to the formation of a pro-fibrotic microenvironment, with immune cells producing an array of cytokines, chemokines, and bioactive factors such as IL-4, IL-6, IL-13, TGF-β, and TNF-α. These factors activate fibroblasts and also shift their phenotype from α-smooth muscle actin-negative (α-SMA-, fibroblast-like) to α-smooth muscle actin-positive (α-SMA+, myofibroblast-like), which, in turn, contributes to ECM deposition and perpetuation of inflammation (57).

Organ fibrosis is a severe condition often leading to fatal outcomes, exemplified in complications of COVID-19 lung injury due to SARS-CoV-2 infection. The massive occurrence of myofibroblasts, coupled with cytokine dysregulation during the cytokine storm, particularly accompanied by elevated levels of IL-6, has systemic effects on the patients (6, 58, 59). Monitoring IL-6 levels in severe COVID-19 cases has been recommended for assessing the health status and adjusting therapeutic protocols to prevent exacerbation of complications (60). Therapeutic interventions targeting the IL-6 signalling pathway, such as anti-IL-6 receptor antibodies tocilizumab or sarilumab, have shown promise in managing these complications (61). In parallel, immune infiltration and the presence of myofibroblasts are common in autoimmune diseases such as the terminal stage of systemic sclerosis or Hashimoto’s thyroiditis (62, 63). The association of systemic sclerosis and other autoimmune diseases with cytokine dysregulation, including hyperproduction of IL-6, has been well-established (64, 65).

Desmoplastic stroma characterises the microenvironment of certain tumours, such as pancreatic ductal adenocarcinoma (PDAC), resembling scar-like fibrosis with numerous myofibroblasts playing a significant role (66, 67, 7). The controlling role of myofibroblasts, particularly in producing IL-6, underscores their potential significance in the stromal desmoplasia of tumours. This intricate interplay between immune response, fibrosis, and tumour microenvironment (TME) highlights the interconnectedness of these biological processes, offering novel avenues for therapeutic exploration.

## Autoimmunity

Autoimmune diseases, characterised by the immune system targeting self-antigens, result in chronic inflammation-mediated tissue injury. Coined by immunologist Paul Ehrlich, the term “horror autotoxicus” captures the phenomenon of immune-mediated self-destruction (Ehrlich, 1900). Despite the immune system’s diverse mechanisms causing tissue damage in autoimmune diseases, fibrosis and impaired function frequently emerge as common features in these deleterious scenarios, demonstrating scleroderma and rheumatoid arthritis as extemporary examples (6).

Scleroderma, also known as systemic sclerosis, is a complex autoimmune disease characterised by fibrotic changes affecting both the skin and visceral organs (68). It leads to increased mortality, particularly due to cardiac disease, pulmonary fibrosis, and pulmonary hypertension. The disease progression is not effectively prevented by current immunosuppressive treatments, as they are only partially successful in halting fibrotic tissue accumulation. Histological examination reveals myofibroblasts as key drivers of fibrosis in scleroderma, and their resistance to apoptosis contributes to abundant collagen overproduction, elevated ECM stiffness, and heightened pro-fibrotic cytokine levels (69, 70). TGF-β (71) and IL-6 (72) are implicated in scleroderma pathogenesis, with IL-6 acting as a pro-fibrotic factor and correlating with disease severity. Inhibition of IL-6 has shown promise in preventing early lung disease progression in patients with systemic sclerosis. Combining different immunotherapies, such as CD47 and IL-6 blockade, has demonstrated efficacy in a murine model, hinting at potential benefits for patients (73).

Rheumatoid arthritis, another autoimmune inflammatory disease, is characterised by joint pain, swelling, and stiffness. Activated mesenchymal cells, particularly fibroblast-like synoviocytes, contribute to pathological tissue repair, leading to pannus formation and joint destruction (74). Extra-articular manifestations, including lung fibrosis, are common in rheumatoid arthritis, and elevated levels of IL-6 are frequently associated with the disease (75). While IL-6 participates in controlling articular and extra-articular pathologies, it is important to note that experiencing the cytokine storm, characterised by elevated IL-6 levels, is relatively rare in these patients (76). However, the autoimmune progression in rheumatoid arthritis can lead to cachexia and severe psychiatric issues, and anti-IL-6 therapy has shown efficacy in treating affected joints, alleviating extra-articular manifestations, stabilising lung fibrosis, and reducing cachexia (77). Notably, drugs such as tocilizumab and sarilumab have demonstrated good efficacy and tolerability in rheumatoid arthritis patients with a poor response to conventional treatments.

## Ageing

Inflammation is a natural process for tissue repair, but chronic inflammation, termed “inflammageing” in ageing individuals, can have adverse effects (78). Immunosenescence, age-related changes in the immune system, and increased cytokine secretion by adipose tissue contribute to chronic inflammation. Elevated levels of IL-6, IL-1, TNF-α, and C-reactive protein in older individuals are linked to higher morbidity and mortality (79), with TNF-α and IL-6 serving as frailty markers. In this context, chronic wounds often stall at the inflammatory stage, where pro-inflammatory cytokines such as TNF-α, IL-1 (α and β), and IL-6 play important roles in signalling immune cell recruitment, angiogenesis, and epithelisation (80). These cytokines, primarily secreted by immune cells but also by epithelial cells, fibroblasts, and endothelial cells, are major components of the senescent secretome. IL-1α, in particular, can stimulate the expression of other cytokines in senescent cells, reinforcing the pro-inflammatory senescence-associated secretory phenotype. The chronic presence of senescent cells in the WME may enhance inflammation and hinder its resolution, contributing to impaired wound healing if the inflammatory stage persists. Understanding this interplay is essential for developing effective interventions in chronic wound management.

In addition to the inflammageing issue, proper wound healing involves transient senescence marked by up-regulated cell cycle arrest proteins (p16, p21, p53) and SASP (81, 82). Studies in mice demonstrate impaired wound healing upon selective elimination of senescent cells, while senescent fibroblasts and endothelial cells, expressing PDGF-AA as part of the senescence-associated secretory phenotype, actively promote wound healing (81). In ageing, the altered senescence response contributes to delayed wound healing, with studies showing improved outcomes in aged mice by inhibiting p21 expression (83). Chronic wounds, such as venous and diabetic ulcers, exhibit senescent fibroblasts, and their presence correlates with decreased healing rates (84). Persistent senescent cells can lead to chronic wounds, emphasising the importance of transient senescence. Additionally, senescent fibroblasts, induced by oxidative stress, regulate the fibrotic response by inhibiting proliferation and matrix synthesis (85), with CCN1 triggering senescence and anti-fibrotic gene expression (86). This complex interplay of senescence dynamics plays a crucial role in regulating the trajectory of wound healing, scarring, and fibrosis.

## Cancer ecosystem: understanding the tumour as an organ

Organogenesis, an intricate ballet of prenatal events encompassing cell proliferation, differentiation, migration, ECM deposition, intercellular interactions, and programmed cell death, orchestrates the correct formation and function of organs. Any deviations during this developmental course can significantly affect the architecture and subsequent function of an organ. In a conceptual shift, malignant solid tumours can be likened to organs, orchestrated by genetically aberrant cancer cells. Their interaction with non-cancer cells, including connective tissue cells, inflammatory cells, and blood vessels, plays a significant role in shaping their aberrant function and subsequent dissemination (87). The connective tissue component, often referred to as the stroma, is a dynamic entity within malignant tumours. It houses fibroblasts producing ECM, growth factors, chemokines, cytokines, matrix metalloproteinases (MMPs), as well as vessels supplying nutrition and oxygen to the tumour (88).

The stroma, far from being a passive bystander, actively participates in the tumour’s dynamic processes. The ECM influences cancer cell adhesion and migration, with proteolytic enzymes remodelling it to create channels facilitating cancer cell migration (11, 89). Furthermore, the stroma acts as a platform for immune cell infiltration into the tumour site. Despite cancer cells expressing numerous neo-antigens due to frequent mutations, the anticancer activity of immune cells is often down-regulated by products of various cell types in the cancer ecosystem. For instance, tumour-associated macrophages may paradoxically support cancer cell growth and spread (90). Accordingly, the intricate dialogue between immune cells and cancer cells opens new horizons in anticancer therapy. Therapeutic manipulation of this dialogue, exemplified by immune checkpoint inhibitors, proves to be a powerful anticancer strategy that enhances the quality of life and life expectancy for many oncological patients (91). Understanding the complexity of the tumour as an organ and deciphering the interplay between its cellular components paves the way for innovative approaches in cancer treatment and management.

Cancer is a genetic disease, grounded in fundamental gene alterations that drive its onset. The molecular scrutiny of individual cancer cells constituting the bulk has unequivocally revealed the heterogeneous nature of tumours, with metastatic cells often displaying significant distinctions from their counterparts in primary tumours (92, 93). Cancer cells, rather than existing in isolation, intricately collaborate with non-cancer cells, which play indispensable roles in fostering cancer cell growth and systemic dissemination. These non-cancer cells actively contribute to shaping a cancer-supporting microenvironment, typically characterised by pro-inflammatory conditions.

Central to the orchestration of tissues and organs are adult tissue stem cells, reliant on specific microenvironments to sustain their stemness. Extending this hypothesis to cancer stem cells suggests that non-cancer cells within the cancer ecosystem and their products actively participate in establishing the niche supportive of cancer stem cell maintenance (94, 95). As cancer progresses, the tumour evolves under the selective pressures exerted by endogenous mechanisms, including the microenvironment, immunity, and exogenous factors such as anticancer therapies (96).

Tumour-associated macrophages (TAM), cancer-associated fibroblasts (CAFs), and various types of lymphocytes are significant components within the cancer ecosystem, each contributing distinctive elements to the intricate milieu (97). Given the scope of this review, our focus will delve into the roles of TAMs and CAFs.

### Tumour-associated macrophages

Macrophages, components of innate immunity originating from the bone marrow, play a critical role in shaping the intricate landscape of the cancer ecosystem. Their plasticity allows for polarisation into distinct subtypes: M1 pro-inflammatory macrophages and M2 alternatively activated macrophages associated with the resolution of inflammation. While M1 macrophages fuel Th1-dependent stimulation, fostering CD8-positive T-cell proliferation and exerting anticancer activity, M2 macrophages orchestrate an immunosuppressive microenvironment and drive tissue remodelling that supports tumour growth and formation of metastasis (98). The shift from M1 to M2 is orchestrated by interleukins IL-4 and IL-13, secreted by various cells within the cancer ecosystem, including cancer cells themselves (99–101). Intriguingly, the recruitment of macrophages to the tumour and their subsequent M2 polarisation fall under the regulatory influence of CAFs (102). Acting as chemical factories, TAMs unleash a diverse array of bioactive factors, including EGF, VEGFA/D, TGF-β, TNF-α, IL-1β, IL-6, IL-24, CXCL1/5/8, CCL2/8, MMP2/9, PDL1, and various non-coding RNAs. These factors exert a multifaceted impact on the tumour ecosystem, stimulating cancer cell proliferation, enhancing invasion, promoting angiogenesis, and concurrently suppressing the immune system’s anticancer activity (103, 104). Unfortunately, the prevailing expression of cancer-supporting factors tends to overshadow the inhibitory signals. For example, it is widely recognised that the inflammatory cytokine IL-1β is a key player in cancer-related inflammation (105). Exploring the therapeutic avenues, the manipulation of TAMs emerges as a suitable target for anticancer interventions, currently in the developmental stage. Propelling M1 cells as carriers of therapeutic cargo to cancer sites presents a promising strategy for the treatment of solid tumours (106).

### Cancer-associated fibroblasts

Traditionally seen as a structural scaffold for organ development, connective tissue and its architects, fibroblasts, have evolved into dynamic contributors to organ homeostasis. These cells produce not only the ECM but also a diverse array of bioactive factors. CAFs often dominate the stromal landscape over cancer cells themselves. In contrast to the historical perception of fibroblasts as uniform entities, recent methodological advancements, particularly single-cell sequencing, have unravelled several fibroblast subtypes. Remarkably, these subtypes exhibit striking inter- and intra-organ heterogeneity and localisze to discrete anatomical positions, offering novel predictions about physiological functions (107). Myofibroblasts, characteriszed by the presence of α-SMA, make a ubiquitous appearance in the pathological scenarios discussed herein - ranging from wound healing and autoimmune disorders to organ fibrosis, including post-infectious conditions. Myofibroblasts, true conductors of the inflammatory orchestra, actively produce inflammation-supporting factors, notably IL-6, thereby shaping a microenvironment conducive to inflammation (6). Unravelling the mysteries surrounding myofibroblasts opens doors to therapeutic possibilities and deeper insights into various pathological conditions.

### Origin of CAFs: Where do they come from?

Previously, the primary origin of CAFs was attributed to the activation of local fibroblasts by TGF-β factors, often involving human endogenous lectin galectin-1 (34); extensive research has revealed a more complex landscape of CAF precursors. Mesenchymal stem cells, emerging from adipose tissue migration (99) or bone marrow recruitment (108), stand out as strong contenders. These mesenchymal stem cells, reprogrammed by cancer cells, acquire CAF functionalities, albeit retaining some characteristics of their original phenotype (109). Despite these observations, the notion of local fibroblasts as the primary source for CAF formation persists, supported by experimental evidence demonstrating that CD26-positive fibroblasts, acting as CAF precursors, exhibit enhanced protumorigenic characteristics compared to their CD26-negative counterparts (110). Further cell types which may also contribute to the CAF pool include stellate cells, adipocytes, mesothelial cells, circulating fibrocytes, pericytes, smooth muscle cells, haematopoietic stem cells, and endothelial cells (111). Multiple factors, such as IL-1β, IL-12, FGF, PDGF, SDF1, HDGF, IFN-γ, and TNF-α, participate in CAF formation from various precursors. However, TGF-β cytokine family factors consistently play an active role, regardless of the precursor source of CAFs (112).

Remarkably, cancer cells undergoing EMT have been identified as potential contributors to the pool of CAFs, a phenomenon observed for example in breast and pancreatic cancers (113, 114). However, a cloud of controversy hangs over this observation, casting doubt on its universality. Experiments injecting human cancer cells into nu/nu mice have unveiled tumours exhibiting a stroma originating from the recipient mouse cells rather than EMT-induced CAFs (115). This discrepancy calls for meticulous examination, necessitating further experiments to untangle the intricacies surrounding the reliability of the utilised model and the true origin of CAFs. Resolving these lingering questions is inevitable for a comprehensive understanding of CAF genesis and its implications in cancer progression.

### Distinguishing CAFs: a challenge in cellular identification resulting from heterogeneity

Vimentin, an intermediate filament, is expressed in fibroblasts, yet its presence in various cell types complicates the task of distinguishing CAFs definitively. Despite efforts, no specific marker exclusive to CAFs has been identified, contributing to the challenge of differentiation. The expression of α-SMA is commonly considered a CAF marker, but its limitations arise from a substantial subset of these cells being negative for this marker. Furthermore, α-SMA is shared with smooth muscle cells, adding to the complexity of the specificity issue (112). CAFs exhibit a diverse array of expressed proteins, including type I collagen, tenascin C, periostin, podoplanin, CCL2, RAB3B, S100A4, CD31, CD74, CD90, DDR2, FSP-1, CXCL12, calponin-1, PDGFR-α, FAP, ASC-1, TGF-β2, and NG2 (112). However, the absence of a singular, distinctive marker underscores the necessity of employing a combination of markers for accurate identification.

In *in vitro* experiments, determining the negativity for proteins associated with other cell types – e.g., keratins (epithelium), CD45 (leucocytes), CD31 (endothelium), and melanocyte markers such as MELAN-A or HMB45 – proves useful for characterising and isolating CAFs (116). The pursuit of more specific markers remains crucial for advancing our ability to identify CAFs in the cellular milieu.

CAFs display heterogeneity, appearing morphologically homogeneous, but further analysis reveals subcategories. MyCAFs, identified by α-SMA positivity, coexist with negative cells. Single-cell analysis delves deeper, identifying distinct subtypes. In experimental settings, dermal fibroblasts in 3D spheroids with melanoma cells differentiate into ECM producers, inflammation-supporting iCAFs, and ID gene-rich cells influenced by TGF-β family proteins, likely representing precursor CAFs (117). Prognostically, a prevalence of inflammation-supporting iCAFs is associated with poorer outcomes (118, 119). Directly isolated tumour CAFs exhibit further subdivision, including antigen-presenting apCAFs expressing MHC class II (120). Their anticancer effects vary with CAF origin (121). These subtypes also differ in metabolic profiles, with iCAFs favouring glycolysis and myCAFs exhibiting elevated tricarboxylic acid cycle markers (122). The functional implications of these metabolic distinctions are actively investigated.

### Functions of CAFs

Summarising the impact of CAFs on cancer cells, they play a multifaceted role in stimulating cancer cell proliferation, EMT, ECM remodelling, anchorage-independent growth, dissemination of metastasis, immunosuppression, angiogenesis, chemoresistance, and radioresistance (123). Notably, iCAFs, particularly through IL-6 and IL-8 production, significantly influence the dialogue within the tumour ecosystem for various malignancies (124, 125). iCAFs’ stimulatory effects, driven by exosomes released by cancer cells, impact hepatocellular, breast and colorectal cancers (126–128). Elevated serum levels of IL-6 and IL-8 are associated with multiple tumour types, including melanoma, lung, oral (squamous), and gastric cancers (129–132). iCAFs also contribute to the pro-inflammatory microenvironment by increasing serum levels of factors such as IFN-α/γ, IL-1β, IL-2, IL-4, IL-5, IL-10, IL-12P70, IL-17A, and TNF-α (133–136).

Several of these factors participate in the premetastatic niche formation, supporting tumour spread (137). Normal dermal fibroblasts from unaffected skin in patients with metastatic melanoma exhibit CAF-like features, suggesting a role in facilitating malignant cell docking (138).

When CAFs express PDL1, they contribute to the immunosuppressive microenvironment, affecting patient prognosis (139). IL-6-induced STAT3 signalling in CAFs is implicated in muscle protein breakdown and cachexia, a severe systemic complication of cancer (140). IL-6, TNF-α, and IL-8 from CAFs influence adipose tissue loss, organ fibrosis, and appetite reduction, associated with cancer wasting and cachexia (141, 142). Additionally, neuropsychological symptoms, including depression in cancer patients, are linked to high levels of IL-6 and TNF-α (143–145).

CAFs secrete ECM molecules, including collagens, elastin, proteoglycans, periostin, hyaluronic acid, and heparan sulphate, influencing cancer cell proliferation and migration (146). Lysyl oxidase produced by CAFs crosslinks collagen strongly via exosomes (147). ECM components such as tenascin-C and fibronectin are up-regulated in tumour stroma, and the signal-specific information promotes tissue stiffness, facilitating cancer growth and dissemination (148).

**Figure 2:**
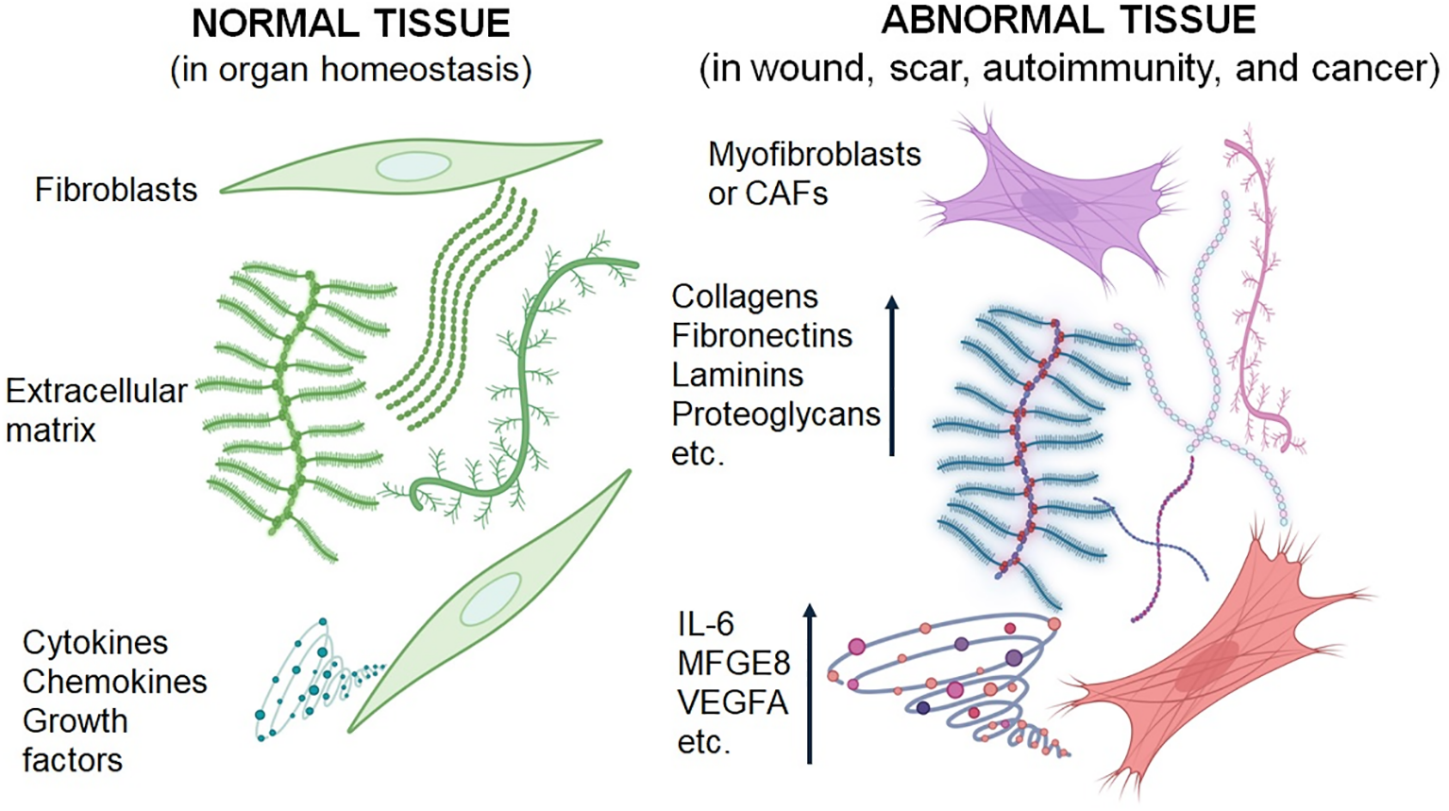
Schematic presentation of the differences between normal fibroblasts and activated fibroblasts associated with wound healing, scar formation, autoimmunity, and cancer. Fibroblasts from pathological conditions exhibit similar characteristics.

Interestingly, the activity of CAFs may not be strictly specific to the tumour type. As we have shown previously, CAFs from one cancer type can influence the phenotype of cells from other cancers (115, 149). Active genes, including *IL6*, *VEGFA*, and *MFGE8*, appear to be common among CAFs across various origins and have a significant impact on cancer cell phenotypes (150). While most CAFs are known to promote cancer progression, local aggressiveness, and metastasis, recent findings indicate that certain subpopulations may exhibit anticancer effects, particularly those associated with MHC II expression and markers such as meflin or CD146 (151–154). Ongoing research aims to unravel the complexities of CAF function and its diverse roles in cancer biology. Differences between normal fibroblasts and those associated with wound healing, hypertrophic/keloid scars, autoimmunity, and cancer are summarised in **Figure 2**. These fibroblasts differ in their expression of α-SMA in some cells, their elevated production of extracellular matrix components, and their secretion of inflammation-supporting factors.

## Paraneoplastic syndromes

The onset of paraneoplastic syndromes may serve as an early warning sign, indicating an underlying health issue. These syndromes typically arise from chronic inflammation, with IL-6 playing a pivotal role and contributing to the dysregulation of blood plasma proteins (155). Many paraneoplastic syndromes exhibit significant biological activity, leading to systemic effects (130). Examples of syndromes not directly linked to cancer therapy are detailed in **Table 2**. Skin, muscles, joints, the nervous system, and endocrine glands appear particularly responsive to inflammation-associated dysregulation associated with cancer development (155). In addition to hormones produced by cancer cells, factors supporting inflammation seem implicated in many paraneoplastic syndromes. These syndromes are not strictly tumour type-specific and are observed in both leukaemias and solid tumours. Furthermore, cancer treatments, including immune checkpoint inhibitors (156), may exacerbate paraneoplastic syndromes, highlighting the potential role of cell disintegration in therapy-related manifestations (157). While the application of anti-IL-6 receptor antibody tocilizumab has a minimal impact on cancer growth, it can reduce the occurrence of paraneoplastic syndromes (158, 159).

**Table 2:** Examples of paraneoplastic syndromes in humans.

Paraneoplastic syndrome	Location	Reference
Paraneoplastic pemphigus	Skin/mucosa	(160)
Chronic urticaria	Skin	(161)
Acanthosis nigricans		(162)
Acrodermatitis paraneoplastica		
Florid cutaneous papillomatosis		
Necrolytic migratory erythema		
Palmoplantar keratoderma		
Lambert-Eaton myasthenic paraneoplastic syndrome	Muscles	(163)
Arthritis	Joints	(164)
Anti-Tif1-gamma autoantibody-positive dermatomyositis	Skin/muscles	(165)
Sensory neuropathy	Peripheral nerves	(166)
Paraneoplastic cerebellar degeneration	Cerebellum	(167)
Limbic encephalitis	Limbic system	
Necrotising myelopathy	Spinal cord	
Endocrine syndrome	Numerous	(168)

## Cancer and the elderly exhibit many systemic similarities

Cancer and ageing share several systemic similarities. Ageing is a complex process influenced by various endogenous and exogenous factors, with chronic inflammation being a hallmark (169, 170). In aged individuals, including humans, there is an elevated level of inflammation-supporting factors in the serum compared to younger counterparts (171–173). High levels of IL-6, both in absolute numbers and when expressed as a ratio to albumin, can predict mortality in seriously ill elderly individuals (174, 175) and may impact cognitive functions in the aged population (176). Similar to cancer cachexia, age-related sarcopoenia in the elderly is associated with increased inflammation-supporting factors (177). The mechanisms of age-related cachexia are not fully understood, but it is hypothesised that chronic inflammation in ageing is linked to the dysregulation of iron metabolism (178). Another explanation for elevated pro-inflammatory activity in the elderly may be differences in intestinal microbiota and intestinal permeability (179). Collectively, these age-dependent differences are referred to as inflammageing, as mentioned above, and can contribute to various age-related disorders (180). Given the negative impact of elevated cytokine levels on the physiological functions of the elderly, reducing inflammation could be a promising avenue to enhance the quality of life for seniors. Some approaches, such as incorporating dietary soy isoflavonoids and proteins, show promise (181). Monitoring inflammation-supporting factors in the serum of polymorbid older individuals is relevant in clinical medicine. The high levels of these cytokines associated with age-related frailty could serve as indicators to identify cancer patients who may not benefit from therapy and could even face a risk of reduced life quality (182).

## IL-6: the key player from wound healing to cancer – therapeutic implications

It has been shown that the formation of wound-healing granulation tissue featuring α-SMA-rich myofibroblasts is a linking point of various pathological conditions such as wound healing, autoimmune inflammation, tissue fibrosis, post-serious infection status, cancer, and age-associated frailty. Central to these processes is the cytokine IL-6 and its signalling pathway (183, 184). IL-6, a glycoprotein of 184 amino acids (MW 23.7kDa), is produced by immune cells and, notably, by non-immune cells such as myofibroblasts. Its function hinges on receptor expression. The transmembrane IL-6 receptor interacts with the signal transducer (gp130) upon IL-6 binding, initiating signalling. However, the IL-6 receptor can also be found in biological fluids. When unoccupied, the IL-6 receptor undergoes enzymatic cleavage from its transmembrane domain, releasing it into the extracellular space. Once interacting with IL-6 and signal transducers, this soluble IL-6 receptor can then dock to the membrane and initiate signalling (185). The immune function, primarily initiation of the immune response, and other functions – spanning from wound healing to cancer – rely on the type of cells that express membrane receptors. This involves docking of the complex of soluble receptors with IL-6 to permissive cells. The non-immune functions encompass epithelial cell proliferation and migration, along with the production of ECM by fibroblasts and neovascularisation.

Given the IL-6’s impact on cancer-related processes, therapeutic modulation of its signalling axis holds promise. A range of drugs, from antibodies to small inhibitors targeting IL-6 signalling, have been developed. These interventions include blocking IL-6 synthesis and its receptor, neutralising IL-6, inhibiting its binding to the IL-6 receptor, and disrupting the interaction of the IL-6 receptor with gp130 (186). Anti-IL-6 therapy, exemplified by the widely used anti-IL-6 receptor humanised monoclonal antibody tocilizumab, is a common practice in rheumatology (187). Moreover, tocilizumab was successfully employed in treating COVID-19-induced cytokine storms in indicated patients (188). A similarly positive effect was also observed with the use of the anti-IL-6 antibody siltuximab, although it was not as commonly utilised as tocilizumab (189). Unfortunately, clinical studies attempting to influence IL-6 interaction with the receptor complex in anticancer treatment were not entirely successful (184). It is suggested that inhibition of Jak/Stat3, which is involved in the signalling pathways of various cytokines, chemokines, and growth factors, including IL-6, could be more effective (190, 186). A combination of anti-IL-6 signalling therapy with other anticancer drugs or drugs inhibiting chronic inflammation in the TME may be more appropriate (190, 191). Another potential approach could involve the use of drugs with dual effects, such as targeting both the IL-6 receptor and mitochondria, representing a promising model for complex therapy (192).

Of note, the potential systemic effects of IL-6 produced by the TME, including CAFs, on cancer wasting, cachexia, and paraneoplastic syndromes are detailed in the chapters “Functions of CAFs” and “Paraneoplastic syndromes”.

## Conclusion

Inflammation influences wound healing, solid cancer progression and spreading, with cytokines, notably IL-6, playing a central role. During wound healing, macrophages and fibroblasts in the granulation tissue are the main contributors to IL-6 production, enhancing wound re-epithelisation. Closure of the wound prompts termination of new granulation tissue deposition, starting the maturation/remodelling phase. Complications such as bacterial infections or foreign body contamination may prolong healing, introducing inflammation and overproduction of myofibroblasts leading to hypertrophic scarring or keloid formation. This scenario mirrors inflammation seen in autoimmune disorders or viral infections (e.g., COVID-19), often resulting in fibrosis, which can be severe and fatal.

**Figure 3:**
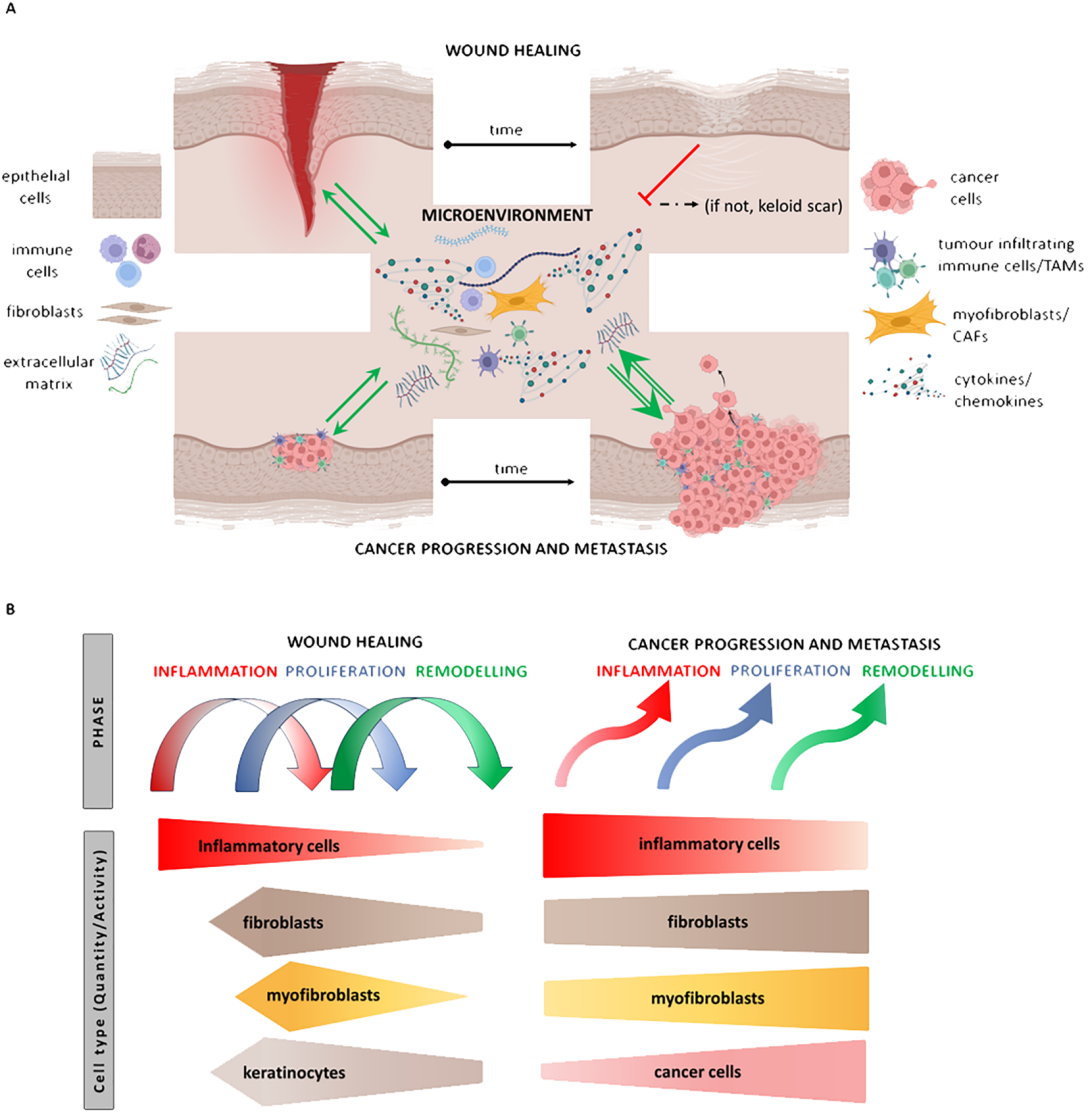
Illustrates the similarities between events in wound repair and cancer. The granulation tissue of the wound/tumour stroma is composed of fibroblasts/cancer-associated fibroblasts (CAFs), macrophages/tumour-associated macrophages (TAMs), and endothelial cells (ECs). Both granulation tissue and stromal elements communicate with normal/malignant epithelial cells through the production of extracellular matrix (ECM) and paracrine secretion of cytokines such as IL-6 and others. While the dialogue between cells of granulation tissue and the proliferating epithelium is switched off after reepithelisation, the unlimited proliferation of cancer cells stimulates further development of stromal tissue. This process leads to the progression of the tumour, its spreading, and subsequent wasting of patients **(A)**. The hierarchy of the steps is disorganised in cancer compared to wound healing **(B)**.

Solid tumours follow a similar narrative, as the cancer stroma, featuring CAFs, resembles the granulation tissue of healing, complete with myofibroblasts, akin to the proliferation phase of wound healing. However, in cancer, the granulation tissue-like stroma does not undergo shutdown, and the dialogue between cancer cells and stromal elements persists endlessly, facilitated by the unlimited proliferation of cancer cells (**Figures 3A, B**). Chronic wounds, autoimmune conditions, and cancer converge in their systemic impact on the patient, leading to frailty, wasting, and cachexia, ultimately compromising the quality of life and contributing to mortality.

The present review weaves narratives under the theme of inflammation, orchestrated by the paracrine secretion of cytokines and chemokines, with IL-6 taking a central role – a ubiquitous cytokine with intricate effects. Wound healing, considered a primary event, is repurposed in autoimmunity and cancer, offering valuable insights into understanding cancer regulation and informing potential anticancer therapies. Although therapies targeting the IL-6 axis have not met all expectations, combining them with other anticancer approaches or employing drugs with multiple targets in cancer ecosystems may prove beneficial in the hopefully near future.
